# Normal butanol fraction of *Polygonum hydropiper* L. flavonoids reduces inflammation caused by PCV2 infections in cell and mouse models

**DOI:** 10.3389/fvets.2025.1539448

**Published:** 2025-02-04

**Authors:** Yu-heng Wei, Shu-mian Zhou, Wen Zhao, Qi Chen, Qiu-hua Wang, Mei-ling Yu, Ying-yi Wei, Ting-jun Hu

**Affiliations:** ^1^College of Animal Science and Technology, Guangxi University, Nanning, China; ^2^Guangxi Zhuang Autonomous Region Engineering Research Center of Veterinary Biologics, Nanning, China; ^3^Guangxi Key Laboratory of Animal Breeding, Disease Control and Prevention, Nanning, China

**Keywords:** porcine circovirus type 2, normal butanol fraction of *Polygonum hydropiper* L. flavonoids, inflammatory response, RAW264.7 cells, model establishment

## Abstract

**Introduction:**

The normal butanol fraction of *Polygonum hydropiper* L. flavonoids (FNB) exhibits significant anti-inflammatory effects. This study investigated FNB's impact on inflammatory responses induced by Porcine circovirus type 2 (PCV2) in cell and mouse models.

**Methods:**

An inflammatory model was established in RAW264.7 cells infected with varying PCV2 concentrations. And assigning both RAW264.7 cells and 108 SPF-grade KM mice to Control, PCV2, Rutin, and various dosages of FNB groups. Inflammatory factors such as Monocyte Chemoattractant Protein-1 (MCP-1), interleukin-6 (IL-6), IL-8, IL-10, Tumor Necrosis Factor-alpha (TNF-α), Reactive Oxygen Species (ROS), and Nitric Oxide (NO) were quantified using ELISA, RT-qPCR and immunohistochemistry.

**Results:**

Results showed that a PCV2 titer of 10^4.5^ TCID_50_/0.1 mL when applied to RAW264.7 cells effectively established an *in vitro* inflammatory model at 12 and 24 h post-infection. Following PCV2 infection, all the inflammatory factors displayed a significant increased both in culture supernatant and intracellular mRNA expression levels (*p* < 0.05 or *p* < 0.01), but these levels were reduced by FNB treatment (*p* < 0.05 or *p* < 0.01). In mouse sera post-PCV2 infection also showed elevated levels of IL-6, IL-8 IL-10, TNF-α, and MCP-1 (*p* < 0.05 or *p* < 0.01). Additionally, mRNA and protein levels for TNF-α, IL-8, IL-10, IL-6, and iNOS rose significantly in lung tissues (*p* < 0.01) but decreased with FNB treatment (*p* < 0.05 or *p* < 0.01).

**Discussion:**

These findings suggest that FNB reduces inflammatory factor production and modulates the inflammatory response triggered by PCV2 infection, potentially enhancing host resistance against it.

## 1 Introduction

Porcine circovirus type 2 (PCV2) infections inflict marked economic damage to the pig production industry (1). It can cause immunosuppression in pigs and is associated with various inflammatory and immune-related diseases (2). Studies reported that PCV2 infection can induce the upregulation of the interleukins (ILs) IL-1β, IL-6, and IL-8. This upregulation may result in excessive inflammatory responses during initial infection stages and attract neutrophils, leading to immunosuppression. Therefore, this affects the normal functioning of the immune response and facilitates the development of PCV2-related diseases (3). Therefore, controlling PCV2 infection and reducing the inflammatory responses it causes is critical to preventing and controlling PCV2-related diseases.

Flavonoids of *Polygonum hydropiper* L. are a class of natural active components extracted from the Polygonaceae plant *Polygonum hydropiper* L., which possess various pharmacological effects, including significant anti-inflammatory activity (4). Studies have reported that *Polygonum hydropiper* L. flavonoids have substantial anti-inflammatory effects by blocking the production of reactive oxygen species (ROS) and nitric oxide (NO) and upregulating the anti-inflammatory IL-10 while preventing the release of IL-1β, IL-6, IL-8, Tumor Necrosis Factor-alpha (TNF-α), and pro-inflammatory factors caused by LPS exposure in Mouse Mononuclear Macrophages Cells (RAW264.7 cells) (5). The RAW264.7 cell inflammation model is a major tool for studying inflammatory responses and evaluating the effectiveness of anti-inflammatory drugs (6). This model allows researchers to investigate the effects of various substances on inflammatory mediators, offering a theoretical foundation and prospective therapeutic techniques for treating inflammation-related disorders (7).

However, there have been no known cases of developing an inflammatory model of PCV2 infection in RAW264.7 cells to date. Furthermore, there is insufficient information about the modulation of PCV2-induced inflammatory responses by the normal butanol fraction of *Polygonum hydropiper* L. flavonoids (FNB). The current study evaluated the effect of FNB intervention on inflammatory responses induced by PCV2 infection in both cellular and animal models. Moreover, this study established a scientific foundation for preventing and treating animal viral infections through plant flavonoids.

## 2 Materials and methods

### 2.1 Reagents

Phosphate-buffered saline (PBS), fetal bovine serum (FBS), and high-glucose DMEM were obtained from Gibco (USA). TRIzol was from Takara Bio (Japan). The commercial ELISA kits were from NeoBioscience Technology Co., Ltd. (China) to analyze IL-10, IL-8, IL-6, Monocyte Chemoattractant Protein-1 (MCP-1), and TNF-α. The ROS Assay Kit was acquired from Applygen (China). The Chinese company Nanjing Jiancheng Bioengineering Institute provided the Nitric Oxide Assay Kit. The HiScript III RT SuperMix for qPCR (+gDNA wiper) and ChamQ Universal SYBR qPCR Master Mix were bought from Vazyme (China). The IL-10, IL-8, IL-6, TNF-α, and iNOS antibodies were acquired from Proteintech (China). The streptavidin-peroxidase (SP) immunohistochemistry (IHC) kit was purchased from Bioss (China).

### 2.2 FNB preparation

The *Polygonum hydropiper* L. was supplied by Tai Hua Pharmaceutical Co. Ltd. Professor Renbin Huang of Guangxi Medical University's School of Pharmacy verified the authenticity of the plant material. Tao et al.'s technique comprised the extraction and purification of the FNB in accordance with the specified protocol (5). According to the HPLC study, FNB had a total flavonoid concentration of 55.3%. Its rutin, quercetin glycoside, and quercetin concentrations were 21.9%, 8.2%, and 20.6%, respectively (5). The solution was prepared in complete DMEM, filtered using a 0.22 μm mesh, and stored at 4°C for later application.

### 2.3 Virus and cells

PCV2 was supplied by Nanjing Agricultural University (China). Using the Reed-Muench assay, PCV2 titers were found to be 10^4.5^ TCID_50_/0.1 mL. RAW264.7 cells were procured from the Shanghai Institute of Biochemistry and Cell Biology at the Chinese Academy of Sciences. Cells were grown in DMEM with 10% heat-inactivated FBS in an incubator at 37°C with 5% CO_2_.

### 2.4 Cell treatment

The grouping and treatment of the model establishment experiment are shown in Table 1, with four replicates per group. RAW264.7 cells (1 × 10^5^ cells/well) were inoculated in 12-well plates. A total of 500 μL of PCV2 dilutions at varying concentrations (MOI 1, 0.1, 0.01, and 0.001) were added to the PCV2 infection groups. At the same time, an equal volume of serum-free DMEM culture media was administered to the Control group. Following a 2-h treatment, the cells were cultured in each well with 1 mL of cell maintenance medium (DMEM culture media containing 2% fetal bovine serum) following three PBS washes. Lastly, samples were collected at various intervals.

**Table 1:** Grouping and treatment of the model establishment experiment.

**Groups**	**Treatment**	**Collection**
Control group	DMEM treated for 2 h	Samples were collected at 4, 8, 12, 24, and 48 h after infection
10^0^ PCV2 group	10^0^ PCV2 dilutions treated for 2 h	
10^−1^ PCV2 group	10^−1^ PCV2 dilutions treated for 2 h	
10^−2^ PCV2 group	10^−2^ PCV2 dilutions treated for 2 h	
10^−3^ PCV2 group	10^−3^ PCV2 dilutions treated for 2 h	

The grouping and treatment for the experiment on the intervention effect of FNB on inflammatory responses are shown in Table 2, with 4 replicates per group. RAW264.7 cells (1 × 10^5^ cells/well) inoculated into 12-well plates. The PCV2, FNB (high-, medium-, and low-dosage), and Rutin groups were administered 10^4.5^ TCID_50_ of the viral solution. In contrast, the Control group was supplemented with equivalent serum-free DMEM culture media. Following a 2-h treatment, the cells underwent three washes with PBS. In the Control and PCV2 groups, 1 mL of cell maintenance solution (DMEM with 2% FBS) was placed in each of the wells. The FNB and the Rutin groups were each supplemented with 1 mL of DMEM containing 80, 40, and 20 μg/mL of FNB and 40 μg/mL of Rutin, respectively, and then placed in an incubator for continued culture. Subsequently, the supernatant or cells were collected from the culture medium at 4, 8, 12, and 24 h for further experiments.

**Table 2:** Grouping and treatment for the experiment on the intervention effect of FNB on inflammatory responses.

**Groups**	**Viral treatment**	**Drug treatment**	**Collection**
Control group	DMEM treated for 2 h	2% FBS-DMEM	Samples were acquired at 4, 8, 12 and 24 hpi
PCV2 group	10^4.5^ TCID_50_ PCV2 treated for 2 h	2% FBS-DMEM	
Rutin40 group	10^4.5^ TCID_50_ PCV2 treated for 2 h	40 μg/mL Rutin	
FNB20 group	10^4.5^ TCID_50_ PCV2 treated for 2 h	20 μg/mL FNB	
FNB40 group	10^4.5^ TCID_50_ PCV2 treated for 2 h	40 μg/mL FNB	
FNB80 group	10^4.5^ TCID_50_ PCV2 treated for 2 h	80 μg/mL FNB	

### 2.5 Mice treatment

The animal experiment referred to Chen's research (8). A total of 108 SPF-grade KM mice, each weighing 20 ± 2 g, comprising an equal distribution of males and females, were acquired from the Animal Experiment Center of Guangxi Medical University (China). Before the experiment, the mice were acclimatized in an environment maintained at 25°C and 65% humidity for 7 days to mitigate environmental stress. Table 3 indicates that the animals were randomly allocated to several groups, namely, Control, PCV2, Rutin (100 mg/kg·BW), and FNB (25, 50, 100, 200, and 400 mg/kg·BW) groups, as well as a highest dose FNB control group (not infected with the virus). Each group contained 12 mice. Mice received daily inoculations of 1 mL PCV2 *via* intraperitoneal injection (0.3 mL/mouse), nasal drops (0.2 mL), and gavage (0.5 mL/mouse) for 3 consecutive days. FNB was gavaged into the mice from day 4 to day 6. On day 7, the animals were euthanized via asphyxiation under the guidelines established by the Guangxi University Animal Ethics Committee. Blood and lung specimens were obtained for subsequent study.

**Table 3:** Mice treatment.

**Groups**	**1–3 (days) all 1 mL/mouse**	**4–6 (days)**
Control group	Physiological saline (PS, 0.9% sodium chloride solution)	PS 0.02 mL/g·BW
FNB400 group	PS	FNB 400 mg/kg·BW
PCV2 group	PCV2	PS 0.02 mL/g·BW
PCV2 + Rutin100 group	PCV2	Rutin 100 mg/kg·BW
PCV2 + FNB25 group	PCV2	FNB 25 mg/kg·BW
PCV2 + FNB50 group	PCV2	FNB 50 mg/kg·BW
PCV2 + FNB100 group	PCV2	FNB 100 mg/kg·BW
PCV2 + FNB200 group	PCV2	FNB 200 mg/kg·BW
PCV2 + FNB400 group	PCV2	FNB 400 mg/kg·BW

Mice received inoculations of either PS or PCV2 through intraperitoneal injection (0.3 mL/mouse), intranasal drops (0.2 mL), and intragastric administration (0.5 mL/mouse) daily for 3 consecutive days. On Days 4, 5, and 6, they received intragastric administration of Rutin or FNB.

### 2.6 Viability of cells

The influence of FNB on RAW264.7 cell viability was assessed using the CCK8 assay. Briefly, a 96-well plate containing a Control group and 7 varying concentrations of FNB groups (12.5, 25, 50, 100, 200, 400, and 800 μg/mL), each with eight repetitions, was inoculated with 5 × 10^4^ cells/mL at 100 μL/well. After adding 100 μL of FNB to each well, the wells were cultured for 24 h in a culture incubator. The growth medium was then taken out 2 h before the culture's end, 100 μL of serum-free DMEM with 10% CCK-8 was placed in each of the wells, and the culture was maintained for 2 h at 37°C with 5% CO_2_. Lastly, a microplate reader was used to detect the OD value at 450 nm, and the CCK8 kit's instructions were followed to determine the maximum safe concentration of FNB on RAW264.7 cells.

### 2.7 Inflammatory cytokines detection

Following the supplier's instructions, the supernatant or serum was collected for the measurement of MCP-1, IL-10, IL-8, TNF-α, IL-6, ROS, and NO levels while employing enzyme-linked immunosorbent assay (ELISA) and related commercial assay kits.

### 2.8 Determination of inflammatory cytokine mRNA levels

As directed by the manufacturer, TRIzol was utilized for RNA extraction from cells and lungs. Reverse transcription was used for transcribing RNA into cDNA. Using the cDNA as the template, RT-qPCR amplification was performed through a SYRB super-mix solution and a CFX96^TM^Real-Time PCR Detection solution (Bio-Rad, USA). Table 4 contains primer sequences for β-actin, MCP-1, TNF-α, IL-10, IL-8, IL-6, and iNOS. The relative mRNA levels, adjusted to β-actin, were determined through the comparative 2^−ΔΔct^ technique.

**Table 4:** List of primers.

**Gene name**	**Sequence (5′to 3′)**	**Product size (bp)**
β-actin	F: TTCCTTCTTGGGTATGGAAT R: GAGCAATGATCTTGATCTTC	183
IL-6	F: TAGTCCTTCCTACCCCAATTTCC R: TTGGTCCTTAGCCACTCCTTC	76
IL-8	F: GGCTTTCCACATTTGAGGACG R: CGTGGCGGTATCTCTGTCTC	77
IL-10	F: TAACTGCACCCACTTCCCAG R: AAGGCTTGGCAACCCAAGTA	89
TNF-α	F: AGCACAGAAAGCATGATCCG R: CTGATGAGAGGGAGGCCATT	107
iNOS	F: CAAGCTGAACTTGAGCGAGGA R: TTTACTCAGTGCCAGAAGCTGGA	164
MCP-1	F: CCACTCACCTGCTGCTACTCAT R: TGGTGATCCTCTTGTAGCTCTCC	76

### 2.9 Immunohistochemistry

For the immunohistochemistry study, lung tissues preserved in 4% paraformaldehyde were embedded in paraffin wax and sliced into 3.5 μm thick pieces. The expression level of the relevant protein was then determined according to the IHC kits.

### 2.10 Statistical analysis

Data were analyzed with SPSS v.22.0, using the LSD test and one-way analysis of variance (ANOVA). Data are shown as mean ± standard deviation. *p* < 0.05 was considered statistically significant.

## 3 Results

### 3.1 Development of a PCV2-induced *in vitro* inflammation model in RAW264.7 cells

**Figure 1:**
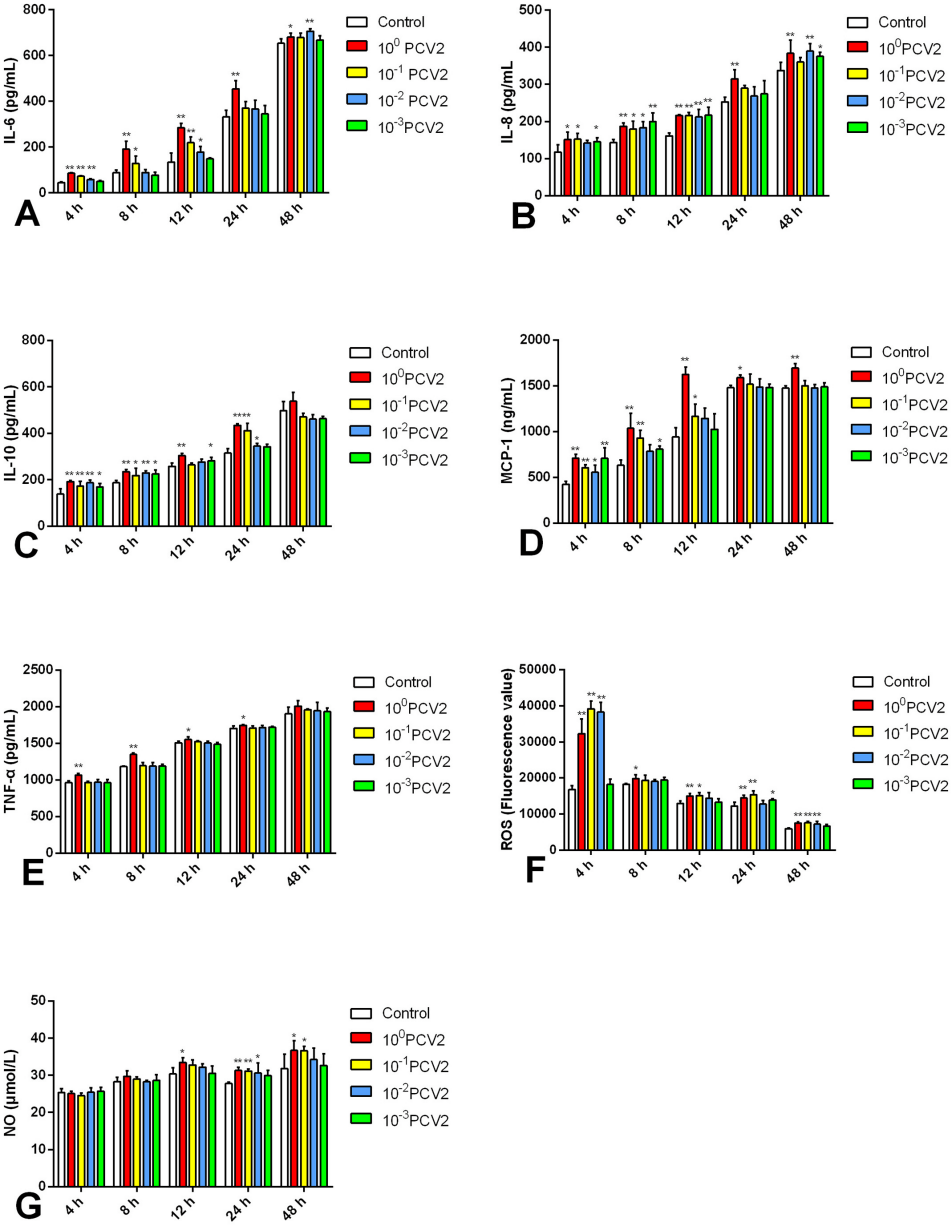
**(A–G)** Effects of PCV2 at different PCV2 dilutions on MCP-1, TNF-α, IL-6, IL-10, IL-8, ROS and NO level (mean ± SD, *n* = 4). ^*^p < 0.05, ^**^p < 0.01 vs. Control group.

Using commercial assay kits, the concentrations of inflammatory cytokines were evaluated *in vitro* in RAW264.7 cells to develop an inflammatory model in RAW264 induced by PCV2 (Figure 1). At 12 and 24 h post-infection (hpi), all measured inflammatory cytokines showed a substantial increase (*p* < 0.05, *p* < 0.01) following infection with 10^0^ PCV2 dilutions in comparison to the Control. This suggests that an *in vitro* inflammatory model can be established using 10^0^ PCV2 dilutions to treat RAW264.7 cells when the PCV2 titer is 10^4.5^ TCID_50_/0.1 mL. Therefore, we selected this condition to carry out subsequent experiments.

### 3.2 FNB reduces the inflammatory cytokines produced by RAW264.7 cells infected with PCV2

**Figure 2:**
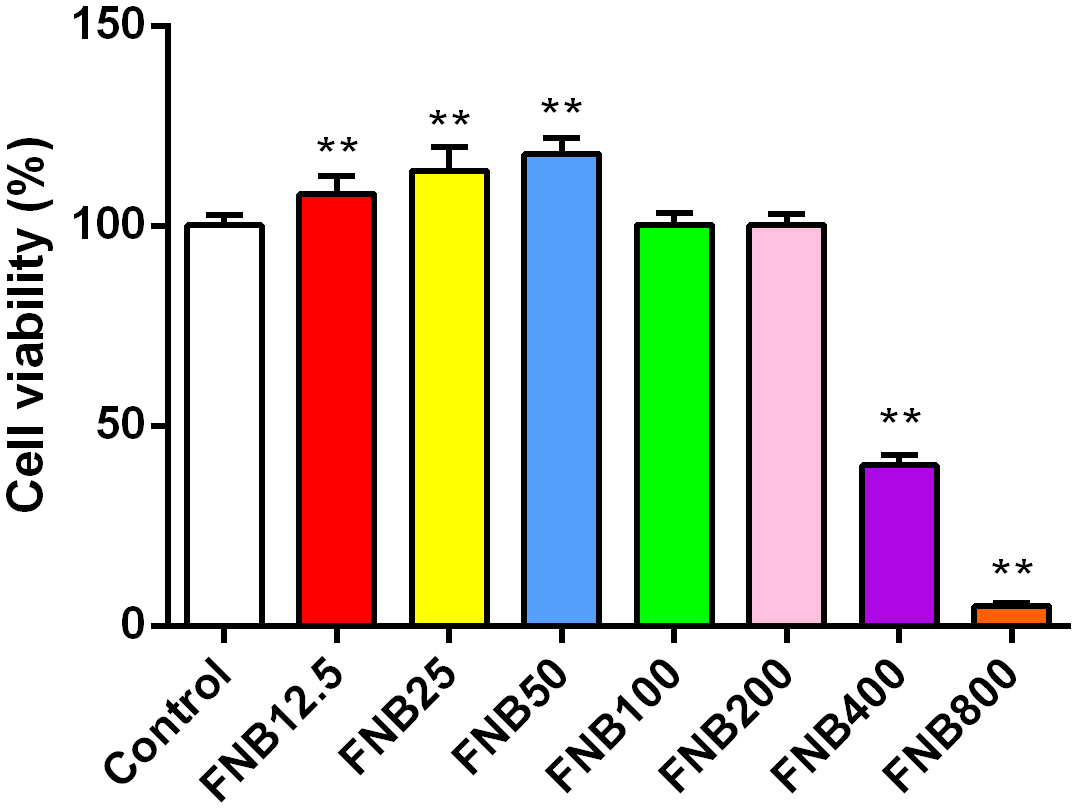
Effects of varying concentrations of FNB on RAW264.7 cell viability (mean ± SD, *n* = 4). ^**^p < 0.01 vs. Control group.

CCK-8 assays were utilized for measuring cell viability. At 24 h post-treatment (hpt), FNB at 12.5, 25, and 50 μg/mL elevated cell viability (*p* < 0.01; Figure 2) while FNB at 100 and 200 μg/mL had no substantial impact (*p* > 0.05). However, FNB at 400 and 800 μg/mL notably reduced viability (*p* < 0.01). Therefore, to examine their intervention effects on inflammatory responses induced by PCV2 infection, FNB concentrations of 20, 40, and 80 μg/mL were finally selected as high, medium, and low dosages, respectively.

**Figure 3:**
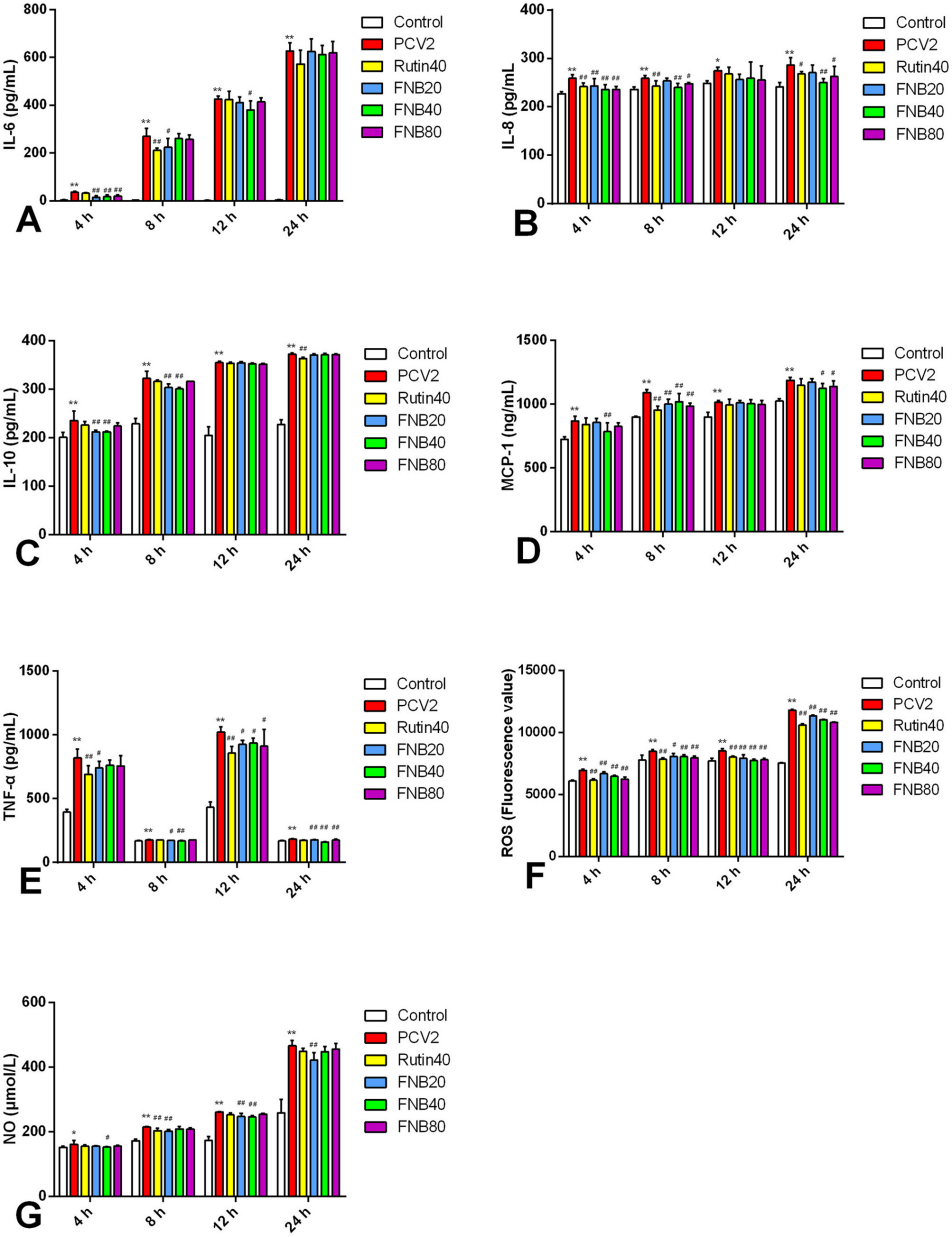
**(A–G)** The impact of FNB as an intervention on inflammatory cytokine levels (mean ± SD, *n* = 4). ^*^p < 0.05, ^**^p < 0.01 vs. Control group. ^#^*p* < 0.05, ^*##*^*p* < 0.01 vs. PCV2 group.

Commercial assay kits were used to explore the intervention impacts of FNB on inflammatory cytokine levels in cells, including MCP-1, IL-8, IL-10, TNF-α, IL-6, ROS, and NO (Figure 3). The efficient development of an *in vitro* inflammatory model was demonstrated by the considerable up-regulation of all evaluated inflammatory factors induced by PCV2 infection relative to the Control (*p* < 0.05, *p* < 0.01).

At 4 or 8 hpt, the PCV2-induced increase in IL-6 and MCP-1 decreased considerably (*p* < 0.01) after exposure to 20, 40, and 80 μg/mL FNB. At 4 and 8 hpt, 40 μg/mL of FNB significantly reduced IL-10 and IL-8 levels (*p* < 0.01). TNF-α and NO levels were substantially lower than those of the PCV2 group after 8, 12, and 24 h following 20 μg/mL therapy (*p* < 0.01 or *p* < 0.05). At 4, 8, 12, and 24 h, ROS levels were significantly reduced (*p* < 0.01) by 20, 40, or 80 μg/mL FNB. An inflammatory response was triggered upon PCV2 infection, seen in raised inflammatory factor levels. Treatment with FNB significantly reduced cell TNF-α, MCP-1, IL-8, IL-6, IL-10, ROS, and NO levels, indicating that FNB could prevent inflammatory responses induced by PCV2.

### 3.3 FNB inhibits inflammatory cytokine mRNA levels in PCV2-infected RAW264.7 cells

**Figure 4:**
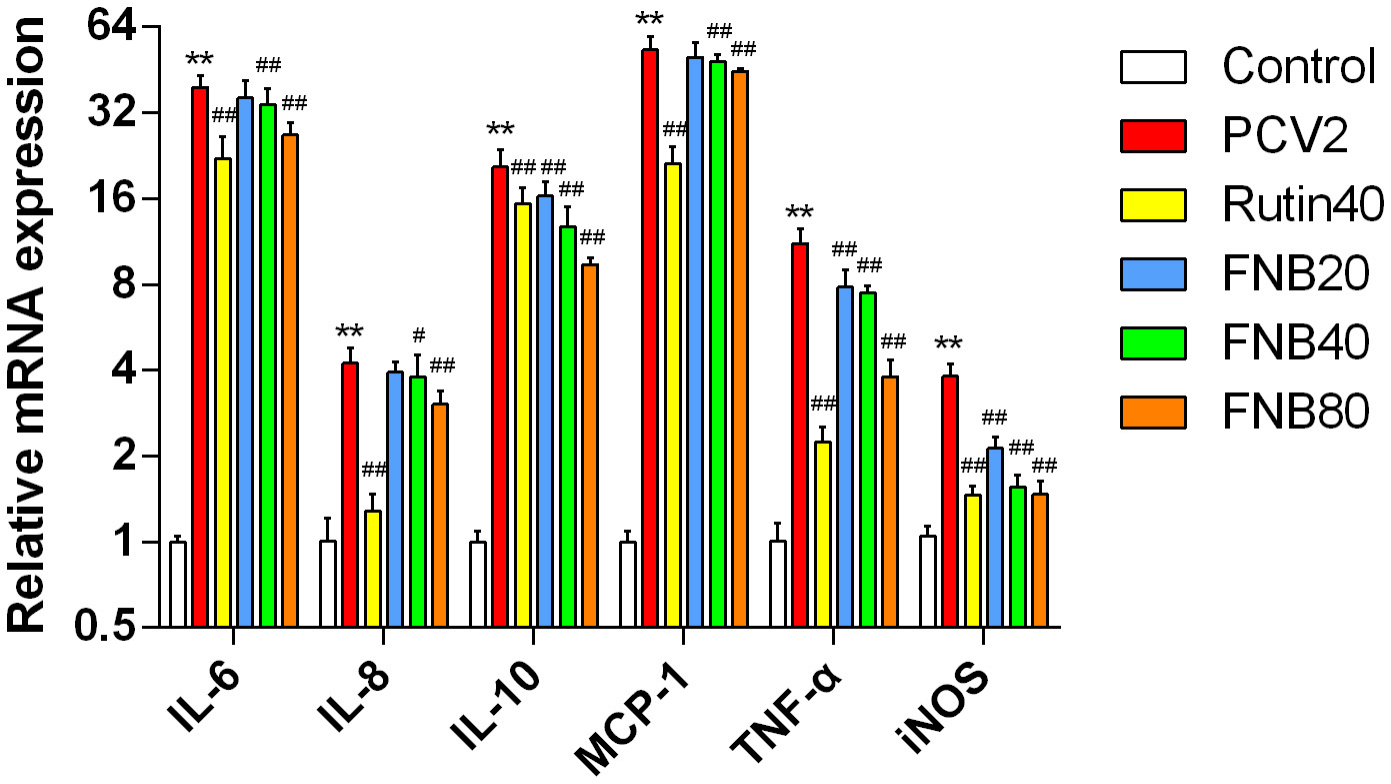
Impact of FNB on inflammatory cytokine mRNA expression levels (mean ± SD, *n* = 4). ^**^p < 0.01 vs. Control group. ^#^*p* < 0.05, ^*##*^*p* < 0.01 vs. PCV2 group.

Using RT-qPCR, the mRNA levels of the cytokines were further investigated. Following infection with PCV2, significant (*p* < 0.01) up-regulation of all six inflammatory factors was observed. Transcription was decreased by 40 and 80 μg/mL FNB administration, as evidenced by lower mRNA levels of MCP-1, IL-8, IL-10, IL-6, TNF-α, and iNOS at 12 hpt (*p* < 0.05, *p* < 0.01) than the PCV2 groups (Figure 4). These findings revealed that FNB could prevent PCV2 from activating genes and that PCV2 produced an inflammatory response by encouraging the expression of associated genes.

### 3.4 Mice infected with PCV2 had lower blood levels of inflammatory cytokines when FNB was administered

**Figure 5:**
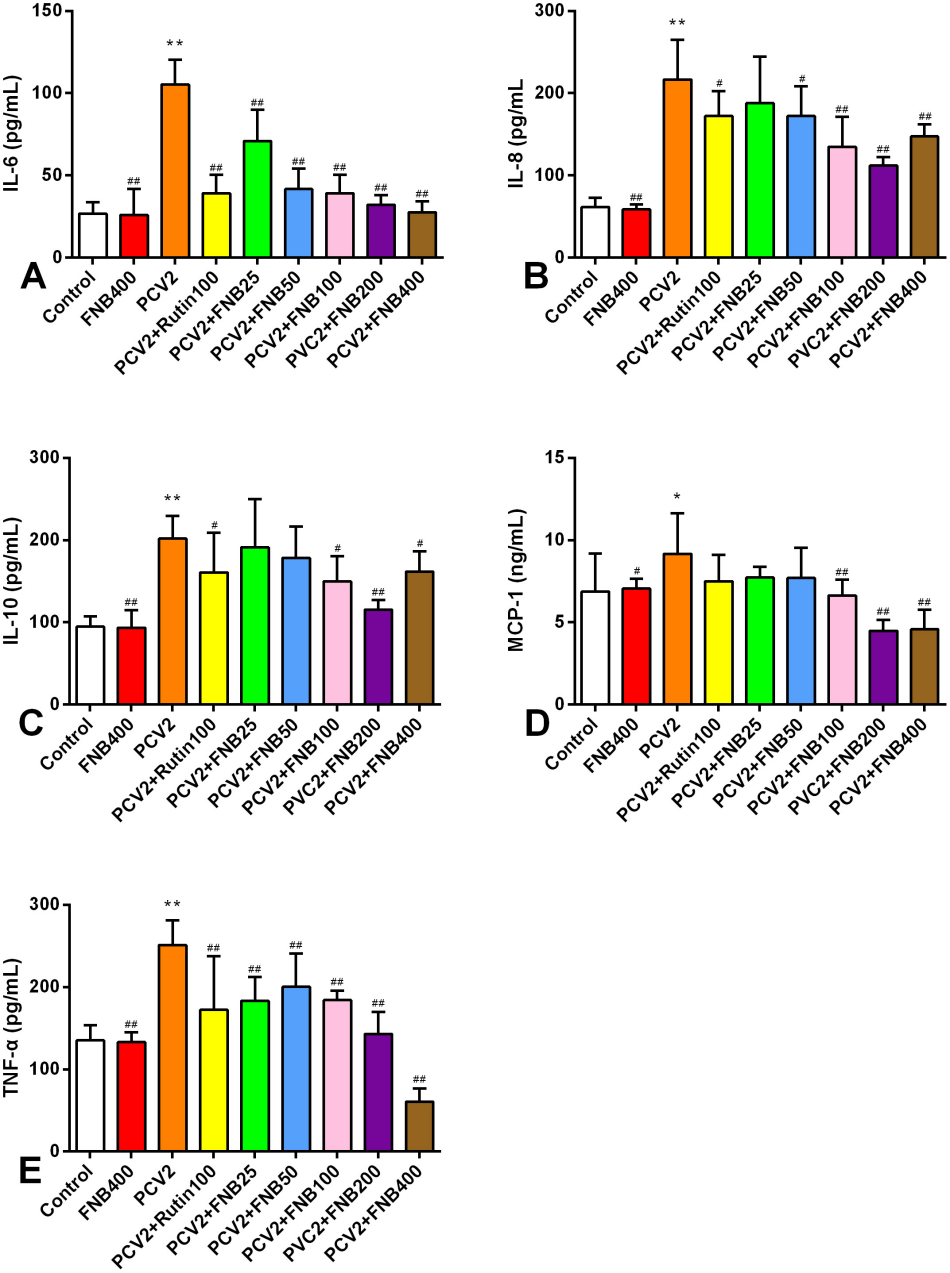
**(A–E)** Effects of FNB on inflammatory cytokine levels in mouse sera after PCV2 infection (mean ± SD, *n* = 6). ^*^p < 0.05, ^**^p < 0.01 vs. Control group. ^#^*p* < 0.05, ^*##*^*p* < 0.01 vs. PCV2 group.

To evaluate the effects of FNB on inflammatory responses *in vivo*, we infected mice with PCV2 and treated them with FNB. The levels of IL-10, IL-6, TNF-α, MCP-1, and IL-8 in mouse sera were assessed by ELISAs. As shown in Figure 5, PCV2 infection significantly increased those inflammatory cytokines (*p* < 0.05 or *p* < 0.01). FNB administration at 100, 200, and 400 mg/kg·BW substantially decreased the increase in those inflammatory cytokines (*p* < 0.05 or *p* < 0.01).

### 3.5 FNB down-regulates inflammatory cytokine mrna levels in mouse lungs following PCV2 infection

**Figure 6:**
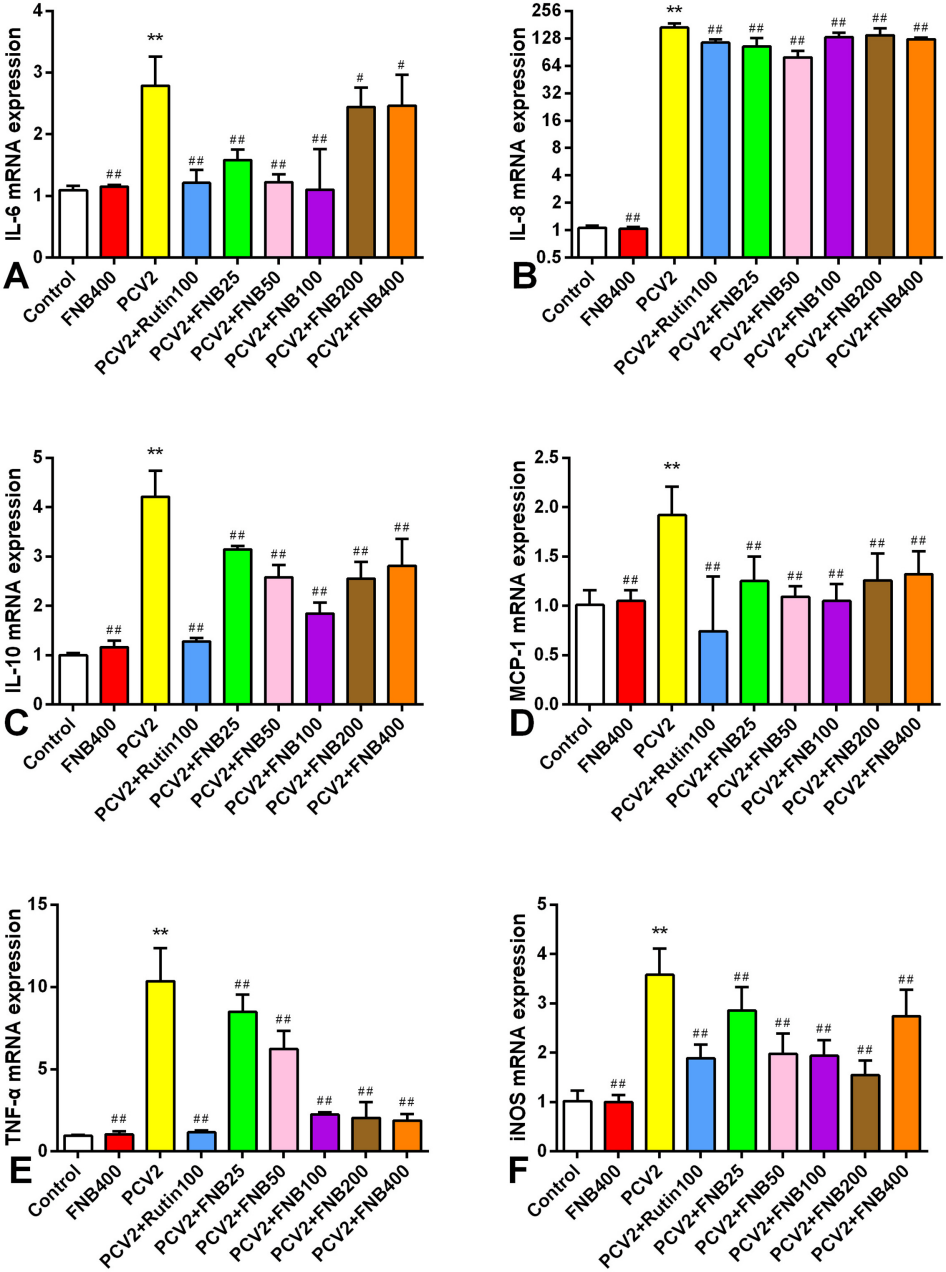
**(A–F)** Impact of FNB on inflammatory cytokine mRNA expression in mouse lungs following PCV2 infection (mean ± SD, *n* = 6). ^**^p < 0.01 vs. Control group. ^*##*^*p* < 0.01 vs. PCV2 group.

We also investigated the intervention effects of FNB on PCV2 infection-induced inflammatory responses at the gene expression level. All inflammatory cytokines listed had significantly higher mRNA expression when infected with PCV2 (*p* < 0.01). Moreover, FNB was able to substantially down-regulate mRNA expression at doses of 25, 50, 100, 200, and 400 mg/kg·BW (Figure 6).

### 3.6 Impact of FNB on inflammatory cytokine protein expression in PCV2-infected mice lungs

**Figure 7:**
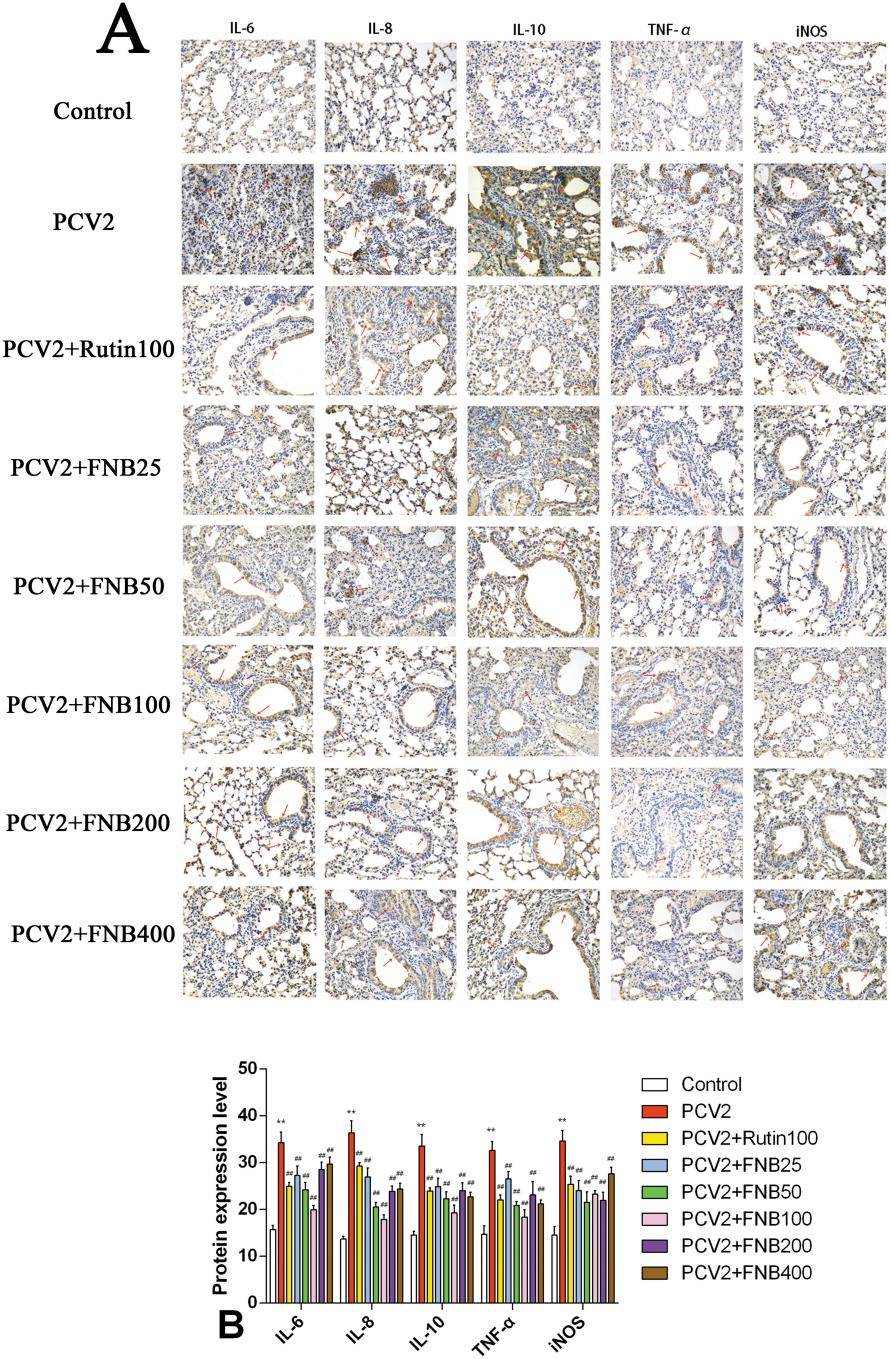
**(A, B)** Effects of FNB on inflammatory cytokine protein levels in mouse lungs following PCV2 infection (mean ± SD, *n* = 6). ^**^p < 0.01 vs. Control group. ^*##*^*p* < 0.01 vs. PCV2 group.

Consistent with the earlier studies, we investigated the intervention effects of FNB on inflammatory responses produced by PCV2 infection using immunohistochemistry methods. The proteins IL-8, IL-10, TNF-α, IL-6, and iNOS were expressed in the lung tissues of both the PCV2 group and the Control group of mice, demonstrating a diffuse distribution in the lungs and localized expression in the cytoplasm of cells, alveolar spaces, blood vessels, and tiny bronchial epithelial cells (Figure 7A). Relative to the controls, the protein iNOS, IL-10, IL-8, TNF-α, and IL-6 expression levels in the lung tissue of PCV2-infected mice were substantially higher (*p* < 0.01). Administration of 25, 50, 100, 200, and 400 mg/kg·BW FNB markedly reduced the protein iNOS, IL-10, IL-8, TNF-α, and IL-6 expressions levels in the alveolar space, fine bronchi, and cell cytoplasm of mice (*p* < 0.01; Figure 7B).

## 4 Discussion

Interleukin-6 is a multifunctional cytokine essential for inflammatory responses and immunological control (9). In reports related to COVID-19, elevated levels of IL-6 are considered a predictive parameter for poor disease progression and the need for invasive oxygen support (10). IL-8 is a crucial inflammatory marker; as a chemokine, it attracts and activates neutrophils, regulates inflammatory responses, and participates in viral replication. It is reported that coronavirus porcine epidemic diarrhea virus utilizes IL-8 to facilitate viral replication by regulating Ca^2+^ flux (11). The levels of IL-8 are significantly elevated in various tumor tissues and patient serum, showing a clear correlation with patient prognosis and tumor staging (12). IL-10 is anti-inflammatory and promotes immune tolerance and regulates immune responses (13). However, in some cases, it may promote the survival of pathogens and the immune escape of tumors, and elevated IL-10 may be associated with the activity of viral infections (14). The CC chemokine family includes MCP-1, often referred to as Chemokine (CC motif) Ligand 2 (CCL2), which is essential for inflammatory reactions (15). It has been found that in COVID-19 patients, raised MCP-1 levels are associated with disease severity, and it can be used to monitor disease progression (16). One crucial pro-inflammatory cytokine necessary for apoptosis, inflammatory reactions, and immunological responses in several physiological processes is TNF-α (17). Inhibitors targeting TNF-α, such as infliximab, adalimumab, and etanercept, have been used clinically to treat multiple inflammatory diseases and have achieved significant therapeutic effects (18). ROS plays a complex and critical role in viral infections and inflammatory responses; excessive production of ROS can exacerbate inflammatory responses and enhance the replication ability of viruses (19). For example, the Porcine reproductive and respiratory syndrome virus utilizes ROS to enhance its replication (20). NO is also an important biological regulatory factor, and changes in NO levels can also serve as an inflammatory marker to determine whether an inflammatory response has occurred (21). NO levels can rise substantially in viral infections, particularly respiratory infections; in COVID-19 patients, for example, high NO levels are positively linked to disease severity (22). In this study, infection of RAW264.7 cells with PCV2 at a titer of 10^4.5^ TCID_50_/0.1 mL for 12 and 24 h resulted in a significant increase of the levels of IL-6, IL-8, IL-10, MCP-1, TNF-α, ROS, and NO. Hence, the *in vitro* inflammatory model was established successfully.

On the other hand, studies have shown that flavonoid compounds possess significant antioxidant, anti-inflammatory, and antiviral properties. They delay the progression of viral infections by alleviating oxidative stress and inflammation by targeting pathways such as NF-κB, MAPK, ERK, and Akt (23). IFN-γ, IL-17, IL-8, IL-1β, IL-6, and TNF-α are inflammatory factors many flavonoids can decrease (24). Sargassum polysaccharide has been shown by Chen et al. to decrease TNF-α, IL-6, IL-1β, IL-8, IL-10, and MCP-1 and mRNA expression levels resulting from PCV2 infection in RAW264.7 cells (25). Chen et al. also showed that *Spatholobus suberectus Dunn*'s total flavonoids may decrease the elevated ROS and NO generation in RAW264.7 cells by PCV2 infection (26). According to a study by Ren et al., in Pseudorabies virus-infected RAW264.7 cells, the ethyl acetate fraction of flavonoids from *Polygonum hydropiper* L. can lower the levels of iNOS, NO, ROS, and other inflammatory markers (27). Ren et al. also found that the ethyl acetate flavonoid fraction from *Polygonum hydropiper* L. can inhibit increases in PCV2-induced TNF-α, IL-1β, IL-8, and NF-κB mRNA levels (28). Thus, our data are consistent with these studies, further showing the intervention impacts of FNB on PCV2-induced *in vitro* inflammatory responses.

According to Wang et al.'s *in vivo* studies, *Panax notoginseng saponins* can lower MPO and iNOS activity significantly while also lowering ROS and NO levels in the splenic lymphocytes of PCV2-infected mice (29). The total flavonoid extract from *Spatholobus suberectus Dunn* has been shown by Fu et al. to lower MPO and iNOS activity, improve thymus and spleen indices in PCV2-infected mice, alter immunological function, and lessen oxidative stress induced by viral infection (30). These findings thus support our previous research, demonstrating the impact of FNB as an intervention on PCV2-induced inflammatory reactions *in vivo*. However, the specific molecular mechanisms underlying the intervention of FNB are still not fully understood. For instance, through which signaling pathways does FNB exert its anti-inflammatory effects, are there any non-coding RNAs involved, and what role do histone acetylation modifications play? These questions will be clarified in further studies conducted in our laboratory.

In conclusion, this study effectively created an *in vitro* PCV2 infection model in RAW264.7 cells and demonstrated that FNB could affect the inflammatory responses induced through PCV2 *in vivo* and *in vitro*. Moreover, the findings offer a foundation for the application of plant flavonoids in the prevention and management of viral infections in animals.

## Data Availability

The original contributions presented in the study are included in the article/supplementary material, further inquiries can be directed to the corresponding author/s.
